# The Effect of Bisphenol A on Puberty: A Critical Review of the Medical Literature

**DOI:** 10.3390/ijerph14091044

**Published:** 2017-09-10

**Authors:** Alberto Leonardi, Marta Cofini, Donato Rigante, Laura Lucchetti, Clelia Cipolla, Laura Penta, Susanna Esposito

**Affiliations:** 1 Pediatric Clinic, Department of Surgical and Biomedical Sciences, Università degli Studi di Perugia, 06129 Perugia, Italy; alberto.leonardi88@gmail.com (A.L.); marta.cofini@gmail.com (M.C.); lauralucchetti@yahoo.it (L.L.); laura.penta@ospedale.perugia.it (L.P.); 2 Institute of Pediatrics, Università Cattolica Sacro Cuore, Fondazione Policlinico Universitario A. Gemelli, 00168 Rome, Italy; drigante@gmail.com (D.R.); cipolla.clelia@gmail.com (C.C.)

**Keywords:** bisphenol A, menarche, puberty, pubertal development, premature thelarche

## Abstract

Many scientific studies have revealed a trend towards an earlier onset of puberty and have disclosed an increasing number of children that display precocious puberty. As an explanation, some authors have considered the global socio-economic improvement across different populations, and other authors have considered the action of endocrine disrupting chemicals (EDCs). Among these, bisphenol A (BPA), an aromatic compound largely used worldwide as a precursor of some plastics and chemical additives, is well known for its molecular oestrogen-like and obesogenic actions. We reviewed the medical literature of the previous 20 years that examined associations between BPA exposure and the age of puberty in humans, considering only those referring to clinical or epidemiological data. Of 19 studies, only 7 showed a correlation between BPA and puberty. In particular, the possible disruptive role of BPA on puberty may be seen in those with central precocious puberty or isolated premature breast development aged 2 months to 4 years old, even if the mechanism is undefined. Some studies also found a close relationship between urinary BPA, body weight, and early puberty, which can be explained by the obesogenic effect of BPA itself. The currently available data do not allow establishment of a clear role for BPA in pubertal development because of the conflicting results among all clinical and epidemiological studies examined. Further research is needed to fully understand the potential role of exposure to EDCs and their adverse endocrine health outcomes.

## 1. Introduction

Endocrine-disrupting chemicals (EDCs) are a group of hormonally active agents to which we are directly or indirectly exposed on a daily basis and that have been widely used in industry for almost a century [1]. They interfere with natural hormones, stimulating or inhibiting their action by binding their receptors or by affecting their synthesis, transport, metabolism, and elimination [2]. As a result, they may cause different kinds of diseases. Concerning EDCs’ toxic effects on health, many studies have confirmed that foetuses, infants, and children are more vulnerable than adults, primarily because of the important role of hormonal balance in both growth and development [3,4]. Moreover, they receive more exposure to these substances than adults, as demonstrated by the higher quantities of EDCs found in urine and blood [5,6].

Bisphenol A (BPA), one of the most studied EDCs, is an aromatic compound largely used worldwide as the precursor of plastics and chemical additives [7]. It is widely used in the production of polycarbonate plastics (very common for transparency, heat resistance, and mechanical properties) and epoxy resins for coating of cans for food and beverages. Humans are exposed to these products mainly through their diet [8] but also dermal absorption and inhalation [9]. Among these, we find EDCs in food and drink packaging, baby feeding bottles, recycling containers, polyvinyl chloride, toys, and dental sealants [10]. EDCs can also be transferred to the baby via the placenta or breast milk [11,12]. Although many countries including Europe (EU Directive 2011/8/EU) have recently drawn up laws restricting the use of BPA in the production of basic necessities [13], this substance is still used on a large scale [14], with more than 3.5 million tons being produced every year [7]. The United States of America is the world’s largest producer, with more than a 1 million tons of BPA produced per year (almost 25% of the global production) [15].

Despite the seriousness of prenatal exposure to EDCs’ toxic effects, the pathological role of BPA becomes more evident during the pubertal period, when neuroendocrine mechanisms, during the development of reproductive organs and the appearance of secondary sexual characteristics, become more vulnerable to environmental stimulation [16,17]. The aim of our review was to find all studies examining the association between BPA exposure and age of puberty in humans. A PubMed search via MEDLINE was undertaken to identify studies matching the following keywords: “bisphenol A” and puberty”, “precocious puberty”, “female puberty”, “male puberty”, “premature thelarche”, “menarche”, and “pubertal development”. The same research was conducted with these keywords replacing the word “bisphenol A” with the most widely used acronym “BPA”. We reviewed all papers published in the last 20 years, and the date of our last search was March 2017. We retrieved 111 studies, excluding animal studies, in vitro studies, BPA chemical analysis studies, and BPA pharmacology studies, and considering only articles that referred to clinical and/or epidemiological data. From the initial collection, we selected 17 scientific papers based on the above-listed criteria; we later added 2 other papers not found in the PubMed database but that were tracked through the bibliographical references. In the end, we split all works into three subgroups: BPA and female puberty, BPA and isolated premature thelarche, and BPA and male puberty.

## 2. Puberty Onset

Puberty is a period of transition between adolescence and adulthood. During this period, full reproductive maturity is reached through the maturation of gonads, permanent genital development, and appearance of secondary sexual characteristics [18]. The harmonic evolution of the phenomena characterizing puberty is closely linked to the activation of the hypothalamic-pituitary gonadotropic cells-gonadal axis [19]. Clinical and experimental evidence has indicated that the hypothalamic-pituitary-gonadal system is in a condition of repression during childhood. 

Mechanisms responsible for the reactivation of this axis are currently unknown. Recently, a neuropeptide known as kisspeptin, encoded by the gene *KISS-1*, and its receptor GPR54 have been revealed as major regulators of the beginning of the reproductive pubertal axis, acting as potent stimulators of pulsatile secretion of the hypothalamic gonadotropin-releasing hormone (GnRH) [20,21]. GnRH neurons have sex steroid receptors through which oestrogen and progesterone can modulate the activity of kisspeptin [22]. Leptin, a hormone produced by adipocytes and an important regulator of both fat mass and gonadal function, is among the factors leading to increased kisspeptin expression during puberty [23]. Other factors involved in controlling the levels of the GnRH network are the transcriptional regulator enhanced at puberty 1 (EAP1) and Ying Yang 1 [24], which operate as inhibitory regulators, and thyroid transcription factor 1 (TTF1) [25], which operates as a stimulator within the GnRH neuronal network.

Other possible puberty regulators may be RFamide-related peptide-3 (RFRP3) neurons, which seem to play an inhibitory action on the GnRH pathway [26,27]: they were first identified in birds and have recently also been found in humans [28,29]. Some authors have suggested that kisspeptin behaves as an accelerator of GnRH, while RFRP3 acts as a brake; through their modulation, the timing of pubertal development is scanned [30]. 

We know that, at the level of the tonic hypothalamic centre, sensitivity to the inhibition of gonadal hormones is greater in childhood and decreases at the onset of puberty. In fact, in childhood, the very small amount of hormones produced by gonads is able to inhibit the release of GnRH and the secretion of gonadotropins [31]. When puberty approaches, there is a progressive reduction of sensitivity of the tonic centre receptor to the sex steroids’ actions. Then, a critical time coinciding with the onset of puberty arrives, during which gonadal hormones are no longer able to inhibit the hypothalamus neurosecretory activity. There is a massively elevated release of GnRH and LH-FSH gonadotropins, and the consequent activation of the gonads leads to an increased circulating level of androgens and oestrogens [32,33].

Oestrogen plays a very important role in sexual differentiation, which occurs at the pubertal stage and is closely linked to several changes in the child’s phenotype: breast growth [34], effect on pubertal growth spurt for both sexes (stimulation of linear growth at low concentrations and closure of the epiphyseal plates and cessation of linear growth at higher concentrations) [35], maturation of sexual characteristics [36], beginning of menses [37], and mutual influence on the hypothalamic-pituitary axis [38]. The last data explain why, in girls with early pubertal signs, elevated levels of oestradiol can be found in blood, even if the mechanism remains unclear [39]. Three different receptors through which oestrogen carries out its functions have been identified: two are nuclear receptors (αER and βER^0^) [40,41], while another one, the most recently discovered, works as a membrane receptor or a cytoplasmic receptor [42]. 

Androgens play a crucial role in male sexual development, and they have two origins: adrenal or gonadal. Adrenal androgens (DHEA, DHEAS, and androstenedione) increase in their secretion at 6–8 years. This moment corresponds with adrenarche, a process that precedes gonadarche by approximately 2 years, which instead represents the true onset of puberty in males. Gonadarche is regulated by increased production of testosterone by the testicular Leydig cells, which in turn are stimulated by activation of the pituitary gland via GnRH pulsatile secretion. Adrenal androgens are responsible for the initial stages of linear growth and pubic hair, changes of tone, appearance of acne, and appearance of hair in the androgen-sensitive areas. However, the most important role in puberty is played by testosterone in collaboration with pituitary hormones, which are implicated in linear growth, increase in testicular volume, appearance of male secondary sex characteristics, and increase in lean body mass [43].

During pre-pubertal age, the levels of endogenous sex steroids are very low, although receptors for oestrogens and androgens are expressed since early childhood [44]. This explains how a small change in levels, even from the outside, may cause a major change in balance of child hormones, such as several pubertal changes [45]. Diseases related to puberty include early and delayed puberty. Precocious puberty is defined as the onset of puberty characteristics in females under the age of 8 years and in males under the age of 9 years [46,47]. The main clinical manifestations of suspicion for precocious puberty in girls are breast development and acceleration of growth rate; other signs include pubic or underarm hair development, acne, and puberty sweating. In boys, an increase in testicular volume (>4 mL) may occur. Late signs may include modifications of penis and scrotum in appearance and size, acne, pubic hair, underarm or facial hair development, and acceleration of linear growth [48]. Pubertal status is conventionally evaluated using Tanner’s staging; for both boys and girls, early puberty is conventionally summed up in that a Tanner stage 2 or higher may occur earlier than expected [49,50].

Precocious puberty is more prevalent in girls (almost 0.2%) than boys (almost 0.05%) [51]. This condition can be caused by early activation of the pituitary gonadostat (central precocious puberty, CPP) and ectopic production of gonadotropins or sex steroids (peripheral precocious puberty, PPP); it may have different origins, such as idiopathic, sporadic, familial, or neurogenic, which is secondary to central nervous system abnormalities [52]. Idiopathic central precocious puberty (ICPP) is the most common cause in females, being characterized by anticipated activation of the hypothalamic-pituitary-ovarian axis [53]. PPP is a rare condition and is usually characterized by the overproduction of sex hormones (oestrogen and testosterone) [54].

A careful remote and family medical history allows for suspicion of forms of early secondary and familiar precocious puberty, respectively. The clinical data listed above are important in association with other information dealing with signs of pubertal development at the pelvic ultrasonography in females (increase in uterine volume, longitudinal diameter >35 mm, uterine body/cervix ratio >1, thickening of endometrial rhyme, or increase in the diameter of the ovaries, longitudinal diameter >3 cm or increase in size of follicles, diameter >9 mm); with detection of testosterone in males (>50 ng/dL) and oestradiol in females (>20 pg/mL, even if for females low estradiol concentrations do not rule out the diagnosis of precocious puberty); or assessment of bone age at the left hand X-ray film (resulting higher than at least 1 year in comparison with the chronological age).

A definite diagnosis is based on the GnRH test: CPP is present when there is an LH blood peak >5 mUI/mL and LH/FSH ratio >0.66 after stimulation with the hypothalamic hormone analogue [55,56,57,58,59]. Magnetic resonance imaging allows discrimination between forms caused by neurologic diseases and those not (including idiopathic forms) [60]. On the other hand, PPP can be diagnosed by the same criteria except the detection of high oestradiol and low gonadotropin blood levels, LH peak <5 mUI/mL, absence of LH/FSH inversion after GnRH stimulation test, and negative cerebral magnetic resonance imaging [61,62]. Pathological effects related to early puberty include insulin resistance, metabolic syndrome, psychosocial disorders, cardiovascular diseases, adult-onset asthma, and breast or reproductive system tumours [63,64,65,66,67].

## 3. Age of Puberty

Many studies have reported a current counter-trend of age for puberty onset in girls compared to the past. The mean onset of thelarche, pubarche, and menarche has decreased over the last decades [49,68]. This trend has been observed both in America [69] and Europe [70]. A study by Biro et al. showed that 16% of girls (with a peak of 23.4% in black non-Hispanic girls) began pubertal development at the age of 7 years, while 30% (with a peak of 42.9% in black non-Hispanic girls) began at the age of 8, well below the previous average [71]. Askglaede highlighted the different average age for onset of secondary sexual characteristics between the beginning of the 1990s and the early years of this decade (in a time interval of approximately 15 years) in Denmark. If the average was 10.8 years old during the 1990s, it dropped 1.02 years between 2006 and 2008 (to 9.86 years old). Girls get their first periods on average 0.29 years earlier (13.13 years) [71]. Genetics, with the A2 polymorphism of the CYP17 gene [72], Oct-2 gene [73], and GPR54/KiSS-1 at the neuroendocrine level [74], and the environment play a role in the definition of puberty onset; in this respect, the general improvement of socio-economic and nutritional conditions of the population only partially justifies this anticipation [75]. Weight-gain, insulin resistance, and metabolic syndrome are well-known factors linked to this condition. Glucose and leptin have been shown to be involved in the control of GnRH secretion, but their role in the timing of puberty remains controversial [75]. What is certain is that obese girls are more likely to have early menarche [76]. Studies have shown that further decreases in the age of onset of pubertal activation signs (breast development in girls and increased testicular volume in boys) have occurred in recent years, despite the maintenance of substantially unchanged standards of life [70]. For this reason, industrialization and daily exposure to environmental pollutants are considered among the causes of this trend because of their ability to interfere with normal bodily function [77,78]. It also explains why the number of studies dealing with EDC and their effects on pubertal development is increasing [37,79]. One of those studies was conducted by Massart et al. in Italy, where in a limited area of northern Tuscany, they have reported a higher proportion of early puberty: the study has raised the suspicion that this growing incidence might be correlated with environmental factors [80].

Sun et al., comparing previous studies, found an anticipation of puberty also in males, but only for white and Hispanic males (not for non-Hispanic black males) [81], although other studies investigating the male pubertal timing trend have presented less reliable data [82]. 

## 4. Bisphenol A (BPA) and Its Oestrogen-Like Role 

As an EDC, BPA is able to alter reproductive, nervous, cardiovascular, and immune systems and interferes with thyroid and pancreatic functions [82]. According to the pharmacological studies, the maximum BPA value to which we can daily expose ourselves as recommended by the European Food Safety Authority is almost 4 mcg/kg/day [83]. However, some studies recommend lower thresholds for exposure without toxicological relevance [84].

Although most of the molecular mechanisms of its effects on the human body are still unknown, BPA is widely studied for its oestrogen-like action. Because of its structural similarity with 17β-oestradiol, many studies have shown that BPA may act as a leading actor in the pathogenesis of several genital and reproductive disorders, such as male and female reproductive tract alterations, female and male infertility, precocious puberty, externalizing behaviour in girls, breast and prostate cancer, menstrual irregularities, and polycystic ovary syndrome (PCOS) [85,86,87,88,89,90]. It is a xenooestrogen and competes with natural oestradiol for oestrogen receptors (αER, βER, and the membrane receptor) [91,92,93]. Furthermore, it may act antagonistically in the presence of oestrogens, such as 17β-oestradiol, and can interfere with oestrogen nuclear receptors [82,94]. The possibility of simultaneously causing early puberty and having a negative role on fertility seems to confirm its dual aspect. This means that it behaves as an agonist or antagonist through the classical or alternative endocrine receptor-dependent signalling pathways [95]. Moreover, its interference with steroid hormones seems to be expressed by antagonizing the action of androgens and thyroid hormones [96,97].

The pathologic mechanism of precocious puberty caused by BPA exposure is related to its oestrogen-like activity, which triggers a positive feedback process for the activity of the GnRH pulse generator, with increased LH and FSH central secretion despite the weak effect as a xenooestrogen [98]. Animal studies have shown the effects of BPA on the hypothalamic-pituitary-gonadal axis through alterations in brain sexual differentiation, higher GnRH secretion, and alterations in the oestradiol-induced LH surge [86]. In a recent study, Mueller et al. showed that BPA is able to induce precocious puberty by stimulating factors implicated in pubertal activation, such as *KiSS1*, and repressing factors inhibiting the onset of puberty, such as *EAP1* and *YY1*. Epigenetic changes may also enable this mechanism. The same study demonstrated the stimulatory effect of BPA on *Dnmt3b*, a DNA methylase, with a possible effect of altered methylation of the genes belonging to the neuronal network of GnRH [99]. 

Another relevant issue is the correlation between BPA and obesity. This correlation was first studied in rats; when exposed prenatally to BPA, they develop dyslipidaemia, hyperleptinaemia, hyperglycaemia, and insulin resistance [100]. In a second step, the obesogen action of BPA in human beings was evaluated and confirmed [101,102]. An interesting study by Trasande et al. showed high levels of urinary BPA in obese children and adolescents [103]. A more recent study by Li et al. found that, in Shangai, girls aged between 9 and 12 years with high levels of urinary BPA were linked to a more than 2-fold risk of obesity [104]. As obesity has an important role in puberty induction, it is not difficult to imagine a possible indirect action of BPA mediated by its interference with glucose and lipid metabolism [105]. Although several hypotheses have been raised, many uncertainties still remain. 

## 5. Bisphenol A (BPA) and Female Puberty

One of the largest and longest clinical studies on pubertal development in girls and exposure to environmental phenols was conducted by Wolff et al. [106]. In 2008, they investigated the exposure to EDCs and pubertal status in a multiethnic group of 192 healthy 9-year-old girls living in New York City. In addition to phytooestrogens, 1,1′-dichloro-2,2′-bis(4-chlorophenyl)ethylene (DDE) and polychlorinated biphenyls (PCBs), they also measured the urinary BPA in this group of subjects. The choice of selecting 9-year-old patients stemmed from the opportunity of having a relatively equal distribution between subjects with puberty signs (53% had breast development predominantly at Tanner stage 2, and 31% had pubic hair development) or subjects with no signs of sexual development. Despite taking into account BMI as a possible modifier of hormonal exposure, they revealed no correlation between the values of urinary BPA and pubertal development [106].

Later, in 2010, Wolff and other colleagues expanded the study by taking into account 1151 American girls among those enrolled in the Breast Cancer and Environment Research Centers (BCERC) [107]. The last group was established by the National Institute of Environmental Health Sciences and the National Cancer Institute that studied environmental chemicals’ influences on the timing of pubertal development in girls. Between 2004 and 2007, they recruited girls aged between 6 and 8 years and followed them throughout puberty. As in the previous study, the authors compared the pubertal stage of these girls and exposure to 3 classes of chemical contaminants: phenols (including BPA), phthalates, and phytooestrogens. The proportion of girls with breast or pubic hair development (30% and 22%, respectively) was lower 1 year after the recruitment compared to the previous study, given the lower age considered. A sample of urine taken at enrolment and another taken 1 year later was also analysed, but no association was found between the level of urinary BPA and pubertal stage [107]. 

In 2015, the same group evaluated 1239 girls taken from BCERC, all of whom were 6–8 years old at enrolment, in order to detect age at first puberty appearance in a 7-year follow-up and correlated this moment with urinary values of 10 different phenols, including BPA [108]. This study estimated a relative risk of early or late puberty linked to phenol exposure. In approximately 85% of girls, signs of pubertal activation (growth of breasts and pubic hair) appeared in the extended follow-up time, but no statistical correlation was found between levels of BPA and early pubertal development. According to this study, there was no correlation between BMI and levels of urinary BPA [108].

A similar study with a smaller group was conducted in Europe by Frederiksen et al., who investigated pubertal status in relationship with urinary levels of 8 hormonally active phenols in 129 Danish children and adolescents aged between 6 and 21, showing the absence of correlation between urinary BPA and pubertal development [109].

Other works have considered the correlation between BPA exposure and age at menarche. McGuinn et al. evaluated this association in girls through the study of the National Health and Nutrition Examination Survey (NHANES) in the period 2003–2010 [110]. All girls analysed were aged 12–19 years, and it was therefore possible to consider the effect of BPA exposure close to the age of menarche (indeed, 72.2% had reached menarche). Age, ethnicity, country of birth, annual household income, level of guardian graduation, and body max index were also considered as potential confounders. In this case, the index of exposure to BPA was represented by urinary BPA. Among the 987 girls that were studied, those with moderate levels of urinary BPA had delayed menarche compared to those with the lowest values. The girls layering for potential confounders did not reach statistical significance, although a common result with other studies was the finding of elevated values of urinary BPA in overweight girls (BM ≥ 85th percentile) who were more likely to have early onset menarche [111].

Another important American study was conducted by Buttke, Sircar, and Martin in 2012 [112]. They considered the link between exposure to certain EDCs and age of menarche in a group of 461 girls aged between 12 and 16 years who had been recruited from Centers for Disease Control and Prevention’s National Health and Nutrition Examination Survey (NHANES)) between 2003 and 2008. In that survey, the study group was given a questionnaire on demographics and related health behaviours, and each subject underwent clinical and laboratory evaluations. Among the 461 girls, it was possible to analyse the levels of urinary BPA in 440 subjects. To obtain the most reliable results, the interaction between urinary phenols and BMI and ethnicity were also considered, as these factors affect the age of menarche. However, regardless of the adjustments made, urinary BPA levels were not significantly associated with age at menarche [112]. 

While these studies did not show a significant correlation between BPA and puberty, different results have been obtained from studies that took into account a population of girls with early puberty. Most of these works were carried out in Asian populations. Durmaz et al. tested the difference in urinary BPA between 28 non-obese Turkish girls affected by idiopathic central precocious puberty and 25 healthy age- and ethnicity-matched control girls [113]. Both were aged 4–8 years. In the control group, the girls considered had no signs of pubertal activation at the time of the study. The level of urinary BPA in the affected group of 26 girls was significantly (*p* = 0.001) higher than that in the control group. The study also took into account the absence of correlation between urinary BPA levels and serum LH, FSH, and oestradiol levels. They did not observe any significant differences between early puberty and use of feeding bottles or nipples. 

Another more recent study by Supornsilchai et al. considered the values of urinary BPA and pubertal stage in 88 Thai girls [114]. The girls were divided into two groups: 41 patients affected by precocious puberty and 47 healthy age-matched controls. Those with signs of early puberty had higher levels of BPA in the urine compared to the control group. In addition, obese or overweight girls with signs of pubertal activation had higher values of urinary BPA compared to normal weight girls and normal pubertal overweight or obese girls. As in other studies, BPA levels were not associated with levels of FSH, LH, or oestradiol [114].

To determine if there was an association between the onset of precocious puberty and levels of EDCs in the plasma, Kwon performed a study in 2011 that confirmed the association between BPA and early puberty [115]. In that case, the determination of exposure to BPA was obtained by dosing the concentration of BPA in serum associated with that of kisspeptin. Both the levels of serum kisspeptin and BPA were significantly higher in the study group, consisting of 31 girls who were diagnosed with central precocious puberty, compared to the control group of 30 healthy age-matched girls. Instead, no correlation was found between serum kisspeptin, BPA, LH, and FSH peak values [115]. 

In 2010, Qiao et al. compared the plasma levels of 3 different phenols (bisphenol A, octylphenol, and 4-nonylphenol) in 110 girls affected by precocious puberty and 100 normal girls [116]. Those values were compared with the volume of the uterus and ovaries and the value of oestradiol. BPA was identified in the blood of 40.9% of girls with precocious puberty compared to 2% of healthy controls. This confirmed that higher values of BPA are found in subjects with early onset of puberty. In such subjects, exposure to this contaminant positively influenced the volume of ovaries and the uterus.

Yum et al. did not agree with the results of previous trials conducted on populations of children with early puberty. In fact, they recruited 150 female patients affected by precocious puberty and 90 control subjects in the Seoul area, measuring plasma values of 10 different EDCs, including BPA [117]. In that study, the levels of BPA of precocious pubertal girls were lower than in healthy children, unlike other compounds, such as monobutyl phthalate (MBP), that was 1.3 times higher in the affected group compared to controls. 

Similar results may be extrapolated from previous works conducted on the Korean population by Lee et al. in 2009 and Han et al. in 2008. In Lee’s study, 30 patients (29 girls and 1 boy) with idiopathic CPP (diagnosed on the basis of clinical and hormonal tests) were included [118]. Levels of serum BPA in this group were not significantly different than those obtained from 30 normal control children. Han et al. came to the same conclusion one year earlier in his study evaluating serum BPA in 100 subjects (50 with precocious puberty and 50 without) [119]. 

In 2016, Buluş et al. were the first to evaluate the BPA and peripheral precocious puberty ratio [120]. Among the Turkish population, they selected 42 patients with idiopathic central precocious puberty (ICPP), 42 patients with peripheral precocious puberty (PPP), and 50 healthy non-obese age-matched girls as controls. The authors did not identify any statistically significant dissimilarity between the mean values of urinary BPA in the three groups. Moreover, they obtained additional data through a questionnaire investigating the possible source of BPA exposure in daily life; the duration of breastfeeding, playing time with plastic toys, and use of creams or cosmetics did not influence the development of early puberty. As a retrospective questionnaire, the answers might be biased. In contrast, both the groups with ICPP and PPP had a higher presence of polyvinyl chloride windows in their houses compared to the control group. In addition, LH, FSH, and oestradiol serum levels were detected in all three groups, but the results were affected by the underlying disease [120]. 

A more recent study that correlated peripheral precocious puberty and urinary levels of BPA was carried out by Lee et al. [121]. They selected 42 patients suffering from central precocious puberty and 40 patients with peripheral precocious puberty, comparing them with a group of 37 age- and sex-matched healthy subjects through the dosage of 84 different sex steroid hormones. The study was born from the idea that the biological changes involved in early puberty may cause changes in the metabolism of human steroids. Furthermore, there may be exposure to EDCs, in particular to bisphenol A. They found that the urinary levels of BPA were slightly higher but not statistically significant in both PPP and CPP groups than in the control group. They also showed that testosterone, androstenedione, androstenediol, 16-alpha-hydroxy-dehydroepiandrosterone, and 5-alpha-androstenedione were higher in both groups with precocious puberty compared to controls. Moreover, the levels of 17-beta-estradiol were higher in subjects with CPP than in the other two groups, and many other oestrogens were slightly increased in both precocious puberty groups. Furthermore, the levels of testosterone, 17-beta-estradiol, and pregnenolone were significantly higher in patients with elevated levels of urinary BPA [121].

Table 1 describes the main epidemiological studies investigating the relationship between BPA and precocious puberty in girls.

## 6. Bisphenol A (BPA) and Isolated Premature Thelarche

As isolated premature thelarche may anticipate a framework of central precocious puberty and result from exposure to xenooestrogens, it is appropriate to consider studies referring to this phenomenon [122,123]. It is well known that physiological breast development accompanies an upsurge in steroid hormones, chiefly oestrogens [124]. Limited clinical or epidemiological studies have evaluated the relationship between the exposure to EDCs and premature thelarche [125]. One of the latest studies investigating BPA was conducted in 2014 on 251 girls aged 4 months to 2 years [126]. Chen et al. selected only patients with at least stage 2 thelarche per the criteria by Tanner and without other signs of pubertal development. The results obtained showed a serum concentration of BPA significantly higher in premature thelarche girls compared to 33 healthy age- and sex-matched controls. Moreover, within the study group, there was a different distribution of BPA values based on the age of girls: the youngest girls had much higher values than the 4-year-old girls. This highlights the possible harmful effect of higher exposure to BPA in the early stages of life [126].

A more recent study contrasts with this result. In 2015, Ozgen et al. measured the levels of urinary BPA in 28 girls with central precocious puberty, 28 girls with premature thelarche, and 22 pre-pubertal children as controls, without identifying any significant differences between the three groups [127]. In their study, they also measured the serum levels of kisspeptin and found higher concentrations in the CPP group but no correlation between the values of BPA and oestradiol. The contrast with Chen’s study may derive from the different ages of patients selected (girls with isolated premature thelarche aged between 5.41 and 8.12 years were analysed); in fact, the previous study found higher values of urinary BPA in the younger girls.

Table 2 summarizes the main epidemiological studies investigating the relationship between BPA and isolated premature thelarche.

## 7. Bisphenol A (BPA) and Male Puberty

Only 2 studies were identified belonging to this specific category. The first work was carried out by Ferguson et al. in 2014, who studied the correlation between prenatal exposure to phthalates and bisphenol A by evaluating maternal urine collected in the third month of pregnancy [128]. They also considered postnatal exposure by sampling the urine of children born to those mothers, with levels of sex hormones and development of secondary sexual characteristics evaluated in 113 boys aged between 8 and 14 years. Prenatal exposure to BPA did not show a clear association with any of the analysed hormones, in contrast to prenatal exposure to phthalates, which seemed to have an inverse relationship with levels of DHEAS and inhibin B and a direct relationship with SHBG (while there was no correlation with the levels of oestradiol, free testosterone, or total testosterone). With regard to exposure during childhood, BPA seemed to be positively associated with levels of SHBG and inversely correlated with total values of testosterone and free testosterone. The most interesting result, probably correlated with the anti-androgenic nature of BPA, was that prenatal exposure to BPA seemed to be associated with a reduced age of onset of adrenarche [128]. 

The second study, conducted by Wang et al. in 2016, evaluated urinary BPA in 671 Chinese boys aged between 9 and 18 years and their pubertal development [129]. The group consisted of young people from three stages of puberty: early puberty, late mid-puberty, and puberty (100% of 13-year-old patients enrolled in the study had begun pubertal development at the time of recruitment and had testicular volume >3 mL, Tanner stage G2). The results showed that moderate levels of urinary BPA seemed to correlate with the early appearance of pubic hair and testicular development, although this latter association was not statistically significant. On the other hand, there was a direct correlation between values of urinary BPA and the delayed appearance of genital development’s final stages. According to this study, BPA exposure in boys anticipated both pubarche and adrenarche [129]. However, it slowed the progression of puberty, especially with regard to the maturation of genitals. Additional information offered by this study was the apparent importance of BMI as not interfering in the relationship between BPA and pubertal development, emphasizing the uncertain role of fat in male puberty in contrast to in females [130,131].

Table 3 shows the main epidemiological studies investigating the relationship between BPA and male precocious puberty.

## 8. Critical Analysis of the Relationship between Bisphenol A (BPA) and Puberty

The first data on BPA and puberty was obtained from studies conducted on animals. A growing number of animal experiments, especially on rats, has highlighted that early puberty onset may be caused by exposure to BPA in the female population [132,133,134,135,136]. Other studies have published results that are inconsistent with these data, in particular those referring to BPA exposure in utero and during breastfeeding [137]. However, other studies on rats have shown no clear correlation with puberty early onset, even among works evaluating postnatal exposure to BPA [138,139]. 

Among the limited number of human studies, similar equivocal results have been found. Only 7 of the 19 scientific articles considered in this review showed a correlation between BPA and pubertal development. Surveys that evaluated female patients with central precocious puberty and isolated premature breast development aged 2 months to 4 years have confirmed that early exposure to EDCs may lead to risk of developing signs of early puberty. However, a direct relationship between BPA levels and age of menarche or pubertal stage has not been found. The reason for the elevated serum or urine values of BPA in children affected by central precocious puberty has not yet been explained. 

The underlying molecular mechanism is not clear: only 2 studies evaluated serum levels of kisspeptin, and both found no association with BPA levels. This might suggest that BPA action on precocious puberty plays a different role towards the activation of kisspeptin’s network. In fact, even if an indirect action of BPA on puberty through interference with the GnRH axis is possible, some studies have ruled out any correlation between blood levels of BPA, FSH, LH, and oestradiol. However, the possibility of identifying elevated levels of BPA in obese or overweight girls is accepted as is the possible obesogenic effect of exposure to BPA and the close relationship between urinary BPA, body weight, and early puberty [101]. Another consideration can be made about Wang’s study in Chinese boys, as a delay in sexual development was found. This result seems to confirm the idea that children with premature pubertal onset undergo a compensatory slowdown in the late-pubertal stage [140,141]. 

The data obtained from surveys are conflicting, perhaps because of the lack of standardization of studies. A further explanation might be the different geographical areas where the epidemiological investigations have been carried out. The most studied populations were in America and Asia, in 5 and 13 papers, respectively. None of the American studies found an effect of BPA on puberty, whereas 7 of the 13 Asian studies found a correlation. The different exposures based on geographic location can probably explain this difference. Furthermore, different pathological expressions resulting from exposure to EDC can be suspected in various human ethnic groups [142]. The association between BPA and early puberty could reflect changes in personal habits, such as familiar and socio-economic conditions or variable pharmacokinetics and metabolism that are difficult to quantify. Zawatski and Lee have advanced another hypothesis for the conflicting results: EDCs show non-monotonic dose-response relationships instead of the fluctuation of hormone levels during pubertal transition; thus, measurements cannot be considered representative of the hormonal condition [43]. 

In the end, the different results of epidemiological studies may be attributed to the various methods used for the determination of BPA exposure. Regarding BPA metabolism, it has a short half-life (5–6 h) in our bodies and is almost completely eliminated after 24 h [143]. Regarding urinary BPA determinations, several studies have demonstrated that single spot samples of urine give an average value of prolonged exposure to such EDCs [144,145]. Teitelbaum stated that single measurements of urinary phenols are fairly representative of 6–12 months of exposure in children [146]. Another advantage of BPA urinary determination is the easy detection (more than 90% of the general population, according to some studies) [147].

## 9. Conclusions

BPA plays a role only in female patients with precocious puberty or premature thelarche and shows no correlation with pubertal development in healthy subjects. Instead, studies on its role in male puberty are still limited and did not provide definitive data. The molecular mechanism underlying its activity as a puberty disruptor is not clear, but a potential interference with metabolism has been hypothesized. However, this is only speculation because the currently available data do not allow establishment of a clear role for EDCs, BPA in particular, on pubertal development. Moreover, the considered populations are often limited, and many final considerations are derived from cross-sectional studies. All findings related to BPA and puberty should therefore be interpreted with caution with respect to these limitations, highlighting the need for more research on the potential role of any EDCs in adverse endocrine outcomes. 

## Figures and Tables

**Table 1:** Overview of epidemiological studies investigating the relationship between bisphenol A (BPA) and precocious puberty in girls.

Reference	Study Area	Type of Study	Study Group	Subjects’ Age	Exposition Measure	Correlation with PP	Other Remarks
*Han* 2008 [119]	Korea	BPA exposition and PP	50 girls with PP + 50 healthy girls		Serum BPA	NO	
*Wolff* 2008 [106]	USA	BPA exposition and pubertal status	192 girls	9 years old	Urinary BPA	NO	
*Lee* 2009 [118]	Korea	BPA exposition and ICPP	29 girls with ICPP		Serum BPA	NO	
*Qiao* 2010 [116]	China	BPA exposition and PP	110 girls with PP + 100 healthy girls		Serum BPA	YES	-higher ovarian and uterus volume in girls with elevated BPA levels
*Wolff* 2010 [107]	USA	BPA exposition and pubertal status	1151 girls from BCERC group	6–8 years old	Urinary BPA	NO	
*Kwon* 2011 [115]	*China*	BPA exposition and CPP	31 girls with CPP + 30 healthy girls	6–8 years old	Serum BPA	YES	-neither correlation between levels of serum kisspeptin and BPA; nor between BPA and LH or FSH
*Buttke* 2012 [112]	USA	BPA exposition and age at menarche	461 girls from NHANES group for years 2003–2008	12–16 years old	Urinary BPA	NO	
*Lee* 2013 [121]	Korea	BPA exposition and CPP/PPP	42 girls with CPP + 40 girls with PPP + 37 healthy girls	7–9 years old	Urinary BPA	YES (not statistically significant)	-testosterone, E2 and pregnenolone values higher in patients with elevated urinary BPA levels; elevated androgens level in PP groups, elevated E2 in CPP group
*Frederiksen* 2013 [109]	Danimark	BPA exposition and pubertal status	129 girls	6–21 years old	Urinary BPA	NO	-the youngest children had higher urinary BPA
*Durmaz* 2013 [113]	Turkey	BPA exposition and ICPP	28 girls affected by ICPP + 25 healthy girls	4–8 years old	Urinary BPA	YES	-no correlation between urinary BPA and serum levels of LH, FSH, estradiol
*Yum* 2013 [117]	Korea	BPA exposition and PP	150 girls with PP + 90 healthy girls	6–12 years old	Serum BPA	NO	
*McGuinn* 2014 [110]	USA	BPA exposition and age at menarche	987 girls from NHANES 2003–2010 data	12–19 years old	Urinary BPA	NO	-moderate urinary BPA levels are correlated with delayed menarche; elevated urinary BPA in overweight girls
*Supornsilchai* 2015 [114]	Thailand	BPA exposition and PP	41 girls with PP + 47 healthy girls	6–8 years old	Urinary BPA	YES	-obese or overweight girls with pubertal activation had higher urinary BPA levels; no association with levels of FSH, LH or estradiol
*Wolff* 2015 [108]	USA	BPA exposition and pubertal status	1239 girls from BCERC Group	6–8 years old	Urinary BPA	NO	-no correlation between BMI and levels of urinary BPA
*Buluş* 2016 [120]	Turkey	BPA exposition and ICPP/PPP	42 girls witch ICPP + 42 patients with PPP + 50 healthy girls	6–8 years old	Urinary BPA	NO	-girls in ICPP and PPP group had higher BMI than control group

**Table 2:** Overview of epidemiological studies investigating the relationship between bishenol A (BPA) and isolated premature thelarche.

Reference	Study Area	Type of Study	Study Group	Subjects’ Age	Exposure Measure	Correlation with PT	Other Remarks
*Chen 2014* [126]	China	BPA exposure and thelarche	251 girls with premature thelarche + 33 healthy girls	4 months to 2 years old	Serum BPA	YES	-Younger girls had higher BPA values than 4-year-old girls
*Ozgen 2015* [127]	Turkey	BPA exposure and thelarche/ CPP	28 girls with CPP + 28 girls with PT + 22 healthy girls	5–8 years old	Urinary BPA	NO	-No correlation between BPA values and serum kisspeptin nor between BPA and oestradiol values

**Table 3:** Overview of epidemiological studies investigating the relationship between bisphenol A (BPA) and male precocious puberty.

Reference	Study Area	Type of Study	Study Group	Subjects’ Age	Exposure Measure	Correlation with PP?	Other Remarks
*Ferguson* 2014 [128]	China	BPA prenatal and postnatal exposure and male puberty	113 boys	8–14 years old	Urinary BPA	NO	-Elevated urinary BPA levels have been found in boys with higher SHBG serum levels; prenatal exposure seems to be associated with early adrenarche
*Wang* 2016 [129]	China	BPA exposure and boys’ pubertal status	671 boys	9–18 years old	Urinary BPA	YES (not statistically significant)	-Exposure to BPA anticipates both pubarche and the adrenarche but delays puberty progression
